# Transcription Factor Hematopoietically Expressed Homeobox Protein (Hhex) Negatively Regulates Osteoclast Differentiation by Controlling Cyclin‐Dependent Kinase Inhibitors

**DOI:** 10.1002/jbm4.10608

**Published:** 2022-02-14

**Authors:** Hisato Watanabe, Hiroyuki Okada, Jun Hirose, Yasunori Omata, Takumi Matsumoto, Morio Matsumoto, Masaya Nakamura, Taku Saito, Takeshi Miyamoto, Sakae Tanaka

**Affiliations:** ^1^ Department of Orthopaedic Surgery, Faculty of Medicine The University of Tokyo Tokyo Japan; ^2^ Center for Disease Biology and Integrative Medicine, Graduate School of Medicine The University of Tokyo Tokyo Japan; ^3^ Department of Orthopaedic Surgery Keio University School of Medicine Tokyo Japan; ^4^ Department of Orthopedic Surgery Kumamoto University Kumamoto Japan

**Keywords:** EPIGENETICS, OSTEOCLAST, HHEX, CELL CYCLE, CYCLIN‐DEPENDENT KINASE INHIBITOR

## Abstract

We investigated the role of hematopoietically expressed homeobox protein (Hhex) in osteoclast development. Trimethylation of lysine 27 of histone H3 at the *cis*‐regulatory element of *Hhex* was maintained and that of lysine 4 was reduced during receptor activator of nuclear factor κB ligand (RANKL)‐induced osteoclastogenesis, which was associated with a reduction of *Hhex* expression. Overexpression of *Hhex* in bone marrow–derived macrophages inhibited, whereas *Hhex* suppression promoted, RANKL‐induced osteoclastogenesis in vitro. Conditional deletion of *Hhex* in osteoclast‐lineage cells promoted osteoclastogenesis and reduced cancellous bone volume in mice, confirming the negative regulatory role of Hhex in osteoclast differentiation. Expression of cyclin‐dependent kinase inhibitors such as Cdkn2a and Cdkn1b in osteoclast precursors was negatively regulated by Hhex, and *Hhex* deletion increased the ratio of cells at the G1 phase of the cell cycle. In conclusion, Hhex is an inhibitor of osteoclast differentiation that is regulated in an epigenetic manner and regulates the cell cycle of osteoclast precursors and the skeletal homeostasis. © 2022 The Authors. *JBMR Plus* published by Wiley Periodicals LLC on behalf of American Society for Bone and Mineral Research.

## Graphical abstract

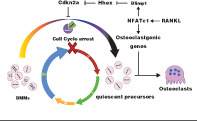

## Introduction

Osteoclasts, the primary cells for bone resorption, are involved in both physiologic and pathologic bone resorption.^(^ ^1^ ^)^ Osteoclast differentiation from monocyte/macrophage‐lineage precursor cells is regulated by two critical cytokines: receptor activator of nuclear factor κB ligand (RANKL) and macrophage colony‐stimulation factor (M‐CSF).^(^ ^2^, ^3^, ^4^ ^)^ Transcription factors such as nuclear factor of activated T cell cytoplasmic 1 (NFATc1), nuclear factor κB (NF‐κB), and c‐Fos play critical roles in osteoclast differentiation.^(^ ^3^, ^5^ ^)^ In addition to positive regulators, several negative regulators of osteoclast differentiation have been identified. Expression of interferon regulatory factor 8 (IRF8) is reduced in response to RANKL, and IRF8 negatively regulates osteoclast differentiation. Targeted disruption of *Irf8* in mice led to severe osteoporosis due to an increased number of osteoclasts, and enhanced bone destruction induced by lipopolysaccharide administration.^(^ ^6^ ^)^ We recently reported that a pioneering transcription factor, PU.1, switches its transcription partners from IRF8 to NFATc1, and alters DNA binding regions during osteoclastogenesis; such features were associated with changes in epigenetic profiles and cell‐type–specific gene expression.^(^ ^7^, ^8^ ^)^ In addition to IRF8, transcriptional factors such as B cell lymphoma (Bcl)6^(^ ^9^, ^10^ ^)^ and MAF bZIP transcription factor (Maf)B^(^ ^11^ ^)^ have been identified as negative regulators of osteoclast differentiation. However, the role of negative regulators and their mechanisms of action have not been fully elucidated compared with positive regulators.

To identify critical negative regulators of osteoclast differentiation, we focused on epigenetic regulation, in particular histone modifications, during RANKL‐induced osteoclastogenesis.^(^ ^12^, ^13^ ^)^ Histone modification plays a crucial role in the regulation of gene expression and cellular differentiation, and trimethylation of lysine 4 of histone H3 (H3K4me3) is associated with transcriptionally active or poised genes. Acetylation of H3K27 (H3K27ac) specifically distinguishes active *cis*‐regulatory elements and antagonizes polycomb repressive complex 2 (PRC2)‐dependent epigenetic silencing by trimethylation of lysine 27 of histone H3 (H3K27me3). We previously reported that RANKL stimulation reduced the H3K27me3 modification of *cis*‐regulatory elements of *NFATc1* by increasing histone demethylase Jumonji D3.^(^ ^13^ ^)^ In the present study, we investigated transcription factors whose histone modification status changed during osteoclast differentiation, and identified the hematopoietically expressed homeobox (*Hhex*, also known as *Prh*) gene as a negative regulator of osteoclast differentiation. Expression of *Hhex* was decreased by RANKL stimulation, and *Hhex* overexpression inhibited and its suppression promoted RANKL‐induced osteoclast differentiation. Interestingly, Hhex regulated the expression of cyclin‐dependent kinase inhibitors (CDKIs) in osteoclast precursors, which play critical roles in the cell cycle progression of osteoclast precursors and their differentiation into mature osteoclasts.

## Materials and Methods

### Reagents

α‐Minimum Essential Medium (α‐MEM), Dulbecco's Modified Eagle Medium (DMEM), and protease inhibitor cocktail were purchased from Nacalai Tesque (Kyoto, Japan). Fetal bovine serum (FBS), penicillin‐streptomycin, and radioimmunoprecipitation assay (RIPA) buffer were purchased from Thermo Fisher Scientific (Waltham, MA, USA). Human M‐CSF was purchased from PeproTech (Cranbury, NJ, USA). Bacteria‐derived soluble RANKL was purchased from Fujifilm Wako Pure Chemical (Osaka, Japan). A tartrate‐resistant acid phosphatase (TRAP) staining kit was purchased from Cosmo Bio (Tokyo, Japan). Polyinosinic–polycytidylic acid (pIpC) and polybrene transfection reagent were purchased from Sigma Aldrich (St. Louis, MO, USA). Lipofectamine 2000 was purchased from Invitrogen (Carlsbad, CA, USA). Cell Counting Kit‐8 (CCK‐8) and Cell Cycle Assay Solution Blue were purchased from Doujindo (Kumamoto, Japan). The anti‐H3K4me3 antibody (polyclonal antibody, rabbit, 39159, RRID:AB_2615077) was purchased from Active Motif (Carlsbad, CA, USA). Anti‐H3K27me3 (polyclonal antibody, rabbit, 07‐449, RRID:AB_310624) and anti‐H3K27ac antibodies (monoclonal antibody, mouse, 05‐1334, RRID:AB_1977244) were from Millipore (Billerica, MA, USA).

### Animal models

*Hhex* ^fl/fl^ (025396) mice on a C57BL/6 background were purchased from Jackson Laboratories (Bar Harbor, ME, USA). *Ctsk* ^Cre/+^ and *Mx‐1* ^Cre/+^ mice were generated as described.^(^ ^14^, ^15^ ^)^ All mice were born and maintained under specific pathogen‐free conditions. Water and food were provided *ad libitum*. All animal experiments were performed with the approval of the Animal Study Committee of Tokyo University Experimental Animal Ethics Committee (P17‐091) and conformed to relevant guidelines and laws. *Hhex* ^fl/fl^ mice were crossed with *CtsK* ^Cre+/−^ mice to generate *CtsK* ^Cre+/−^, *Hhex* ^fl/fl^ mice (*Hhex* ^ΔOC/−^). *Hhex* ^fl/fl^ mice were crossed with *Mx‐1* ^Cre+/−^ mice to generate *Mx‐1* ^Cre+/−^, *Hhex* ^fl/fl^ mice (*Hhex* ^MxCre/−^). pIpC (12.5 μg/g body weight) was administered to 12‐week‐old mice intraperitoneally dissolved in phosphate‐buffered saline (PBS) once a week at least 3 weeks prior to their use in experiments. *Hhex* ^fl/fl^ mice were used as controls for *Hhex* ^ΔOC/−^ and *Hhex* ^ΔMxCre/−^ mice.

### In vitro osteoclastogenesis and osteoclastogenesis assay

To prepare bone marrow macrophages (BMMs), murine bone marrow cells were cultured in α‐MEM with 10% FBS, 1% penicillin‐streptomycin, and 100 ng/mL of M‐CSF for 5 days as described.^(^ ^7^ ^)^ BMMs were used as osteoclast precursors and further cultured in the presence of 10 ng/mL of M‐CSF and soluble RANKL (sRANKL) (concentrations were tailored to each experiment) for 3 days to generate mature osteoclasts. Cells were fixed with 10% Formalin Neutral Buffer Solution for 5 minutes and stained with a TRAP staining kit. TRAP‐positive cells with more than five nuclei were counted as osteoclasts.

### Quantitative reverse‐transcription PCR analysis

Total RNA was extracted with a Direct‐zol RNA Microprep kit (Zymo Research, Irvine, CA, USA) and reverse‐transcribed using a ReverTraAce quantitative reverse‐transcription PCR (RT‐qPCR) Master Mix (Toyobo, Osaka, Japan) to produce single‐stranded cDNA according to the manufacturer's protocol. PCR was performed on Thermal Cycler Dice® Real Time System III (Takara Bio, Shiga, Japan) using THUNDERBIRD SYBR qPCR Mix (Toyobo) according to the manufacturer's instructions. Amplification data were quantified using the standard curve method. Detected signals were confirmed to be specific by a dissociation protocol. All reactions were performed in triplicate.

Primer sequences used for real‐time RT‐PCR analysis were as follows:

*Hhex*‐F: 5′‐ GTTTCAGAATCGCCGAGCTAAAT‐3′,R: 5′‐ CTGCTCACAGGAAGTGTCCAAA‐3′;*Cdkn2a*‐F: 5′‐CTGAATCTCCGCGAGGAAAGC‐3′,R: 5′‐GCCCATCATCATCACCTGAATCG‐3′;*Nfatc1*‐F: 5′‐CAAGTCTCACCACAGGGCTCACTA‐3′,R: 5′‐GCGTGAGAGGTTCATTCTCCAAGT‐3′;*Blimp1*‐F: 5′‐TTCTTGTGTGGTATTGTCGGGACTT‐3′,R: 5′‐TTGGGGACACTCTTTGGGTAGAGTT‐3′;*beta‐Actin*‐F: 5′‐CAGCCTTCCTTCTTGGGTATG‐3′,R: 5′‐AGGTCTTTACGGATGTCAACG‐3′;*Acp5*‐F: 5′‐GACCACAACCTGCAGTATCTTC‐3′R: 5′‐CATAGTGAAACCGCAAGTAGCC‐3′*Oscar*‐F: 5′‐ATCAGTTTCGAAGGTTCTGGC‐3′R: 5′‐CTGCTGTGCCAATCACAAGTA‐3′*Cdkn1b*‐F: 5′‐CAGACGTAAACAGCTCCGAATTA‐3′,R: 5′‐TCAGTGCTTATACAGGATGTCCA‐3′;*Cdkn1a*‐F: 5′‐AGAACGGTGGAACTTTGACT‐3′,R: 5′‐GAGTGCAAGACAGCGACAAG‐3′.*Tp53*‐F: 5′‐TTCTCCGAAGACTGGATGACTGC‐3′,R:5′‐CGTCCATGCAGTGAGGTGATG‐3′

### Immunostaining

After osteoclastogenesis was induced by soluble RANKL and M‐CSF for 2 days, cells were washed with PBS, fixed with 4% (wt/vol) paraformaldehyde for 15 minutes at room temperature, and subsequently permeabilized for 5 minutes with 0.1% (vol/vol) Triton X‐100. Next, cells were rinsed twice with PBS and blocked in 10% (vol/vol) normal goat serum for 1 hour. After blocking, cells were washed with PBS, followed by staining with primary antibody or rhodamine‐conjugated phalloidin (1:200) for 1 hour at room temperature. After washing with PBS, 0.1 μg/mL DAPI (Sigma‐Aldrich) was added for nuclear staining. Fluorescence images were obtained using confocal microscopy (Keyence, Osaka, Japan).

### Western blotting

Cells were harvested after washing with ice‐cold PBS and then lysed in RIPA buffer containing protease inhibitor cocktail. Whole‐cell lysates were subjected to sodium dodecyl sulfate polyacrylamide gel electrophoresis (SDS‐PAGE) and transferred onto polyvinylidene fluoride membranes (Millipore). After blocking, the membranes were incubated with the following primary antibodies: anti‐Hhex (ab34222; Abcam, Cambridge, UK), anti‐FLAG (F3165; Sigma‐Aldrich), anti‐GFP (A‐11122; Thermo Fisher Scientific), and horseradish peroxidase (HRP)‐conjugated beta actin (HRP‐60008; Proteintech Technology, Chicago, IL, USA). Antibody detection was accomplished using HRP‐conjugated secondary antibodies (W4021 or W4011; Proteintech) and chemiluminescence signals were developed with Chemi‐Lumi One Super (02230; Nacalai Tesque). Signals were detected and analyzed by a CL1000 Imaging System iBright (Invitrogen).

### Plasmid construction and retrovirus transfection

For *Hhex* overexpression, the full‐length coding sequence of mouse *Hhex* (NM_008245) was cloned from mature osteoclasts by digestion with *EcoRI* and *XhoI*, and subcloned into a retroviral vector (pMX‐IRES‐Puro). The retroviral vector pMX‐Cre has been described.^(^ ^10^ ^)^ For the chromatin immunoprecipitation sequencing (ChIP) assay, mouse *Hhex* was cloned into the pMX‐Puro plasmid, whereby the C‐terminal end of the gene contained a 3 × FLAG sequence. To generate retroviral particles, retroviral vectors were transfected into the packaging cell line 293GPG using Lipofectamine 2000 according to the manufacturer's protocol. Supernatants containing retroviruses were collected after 24 hours of transfection. Retroviral particles were used for infection after concentration by centrifugation at 6000*g* for 16 hours. For retroviral infection, BMMs precultured with M‐CSF were incubated with retrovirus particles in the presence of 10 ng/mL of M‐CSF and 3 μg/mL of polybrene for 6 hours, followed by culture overnight in the presence of 10 ng/mL of M‐CSF. To select transduced BMMs, cells were detached once with trypsin/EDTA (Sigma‐Aldrich) and cultured in α‐MEM containing 10% FBS, 1% penicillin‐streptomycin, 10 ng/mL of M‐CSF, and 3 μg/mL of puromycin for 2 days. After puromycin selection, BMMs were used to generate osteoclasts or for ChIP experiments.

### Bone phenotype analysis

Micro–computed tomography (μCT) scanning was performed using a InspeXio SMX‐100CT system (Shimadzu, Kyoto, Japan). Scanning was conducted at 90 kV and 40 μA, and the resolution of a single CT slice was 1024 × 1024 pixels. For bone morphology measurements, a range of 1 mm from the epiphyseal line distal to the femur was analyzed. Three‐dimensional microstructural image data were reconstructed and structural indices were calculated using TRI/3D‐BON software (RATOC Systems, Osaka, Japan).

### Histomorphometric analysis

Histomorphometric analysis was performed at magnification ×400 on undecalcified sections from the secondary spongiosa area of the proximal tibia as described.^(^ ^16^ ^)^ For analysis of bone mineralization, mice were injected intraperitoneally with 8 mg/kg calcein 5 days and 2 days before they were euthanized. The eroded surface (ES), osteoclast surface (Oc.S), and osteoblast surface (Ob.S) were measured. The eroded surface/bone surface ratio (ES/BS), osteoclast surface/bone surface ratio (Oc.S/BS), osteoblast surface/bone surface ratio (Ob.S/BS), and mineral apposition rate (MAR) were calculated. The balance of bone metabolism in the proximal tibia as the % ratios of osteoid surface/bone surface (OS/BS), ES/BS were calculated.

### ChIP

ChIP sequencing (ChIP‐seq) was performed as described.^(^ ^7^ ^)^ In brief, cells were fixed with 1% formaldehyde at room temperature and then neutralized with glycine. Next, cells were harvested, resuspended, sonicated, and then incubated with protein A/G beads that had been preincubated with 4 to 10 μg of the required antibody. Immunoprecipitates were washed and reverse crosslinked. DNA was purified with a PCR purification kit (Qiagen, Hilden, Germany). DNA libraries were prepared for sequencing using the standard Illumina protocol (Illumina, San Diego, CA, USA). Purified DNA was used for cluster generation and sequencing on a cBot Cluster Generation system and Genome Analyzer IIx system (Illumina) according to the manufacturer's instructions. ChIP‐seq reads were mapped onto a mouse reference genome sequence (mm9) using Bowtie software (version 1.1.2; http://www.bowtie‐bio.sourceforge.net/).

### Cell proliferation assay and cell cycle analysis

The cell proliferation rate was assessed by CCK‐8 assay. The absorbance of light at 450 nm was measured by a microplate reader to indirectly reflect the number of viable cells. Each group of BMMs was seeded in a 96‐well plate at a density of 2.5 × 10^4^ cells/well in 100 μL medium containing 10 ng/mL M‐CSF. CCK‐8 was added to each well and incubated at 37°C for 2, 24, 48, 72, or 96 hours. The absorbance of the supernatant of each culture was measured at 450 nm using a microplate reader.

Cell‐cycle analysis was performed using Cell Cycle Assay Solution Blue (Doujindo). Briefly, BMMs from *Hhex* ^fl/fl^ and *Hhex* ^MxCre/−^ mice were seeded in six‐well plates. After 24 hours of culture, cells were collected and suspended in 500 μL of PBS. Next, 5 μL of Cell Cycle Assay Solution Blue was added to each cell suspension, and incubated at 37°C for 15 min. The cell‐cycle status of cells was investigated using Cytoflex (Beckman Coulter, Brea, CA, USA); cells at G0/G1, S, and G2/M phases were analyzed using Kaluza analysis software (Beckman Coulter).

### Statistical analysis

Statistical tests for each assay were chosen based on their appropriateness for the assay. Each series of experiments was repeated at least three times. All statistical calculations were carried out using Graphpad Prism (GraphPad Software, San Diego, CA, USA). Statistical analyses were performed using unpaired Student's *t* test. A *p* value of <0.05 was considered statistically significant. Statistical methods and values including all *p* values are described in the figure legends.

## Results

### Hhex gene expression is downregulated in an epigenetic manner during osteoclastogenesis

**Fig. 1:**
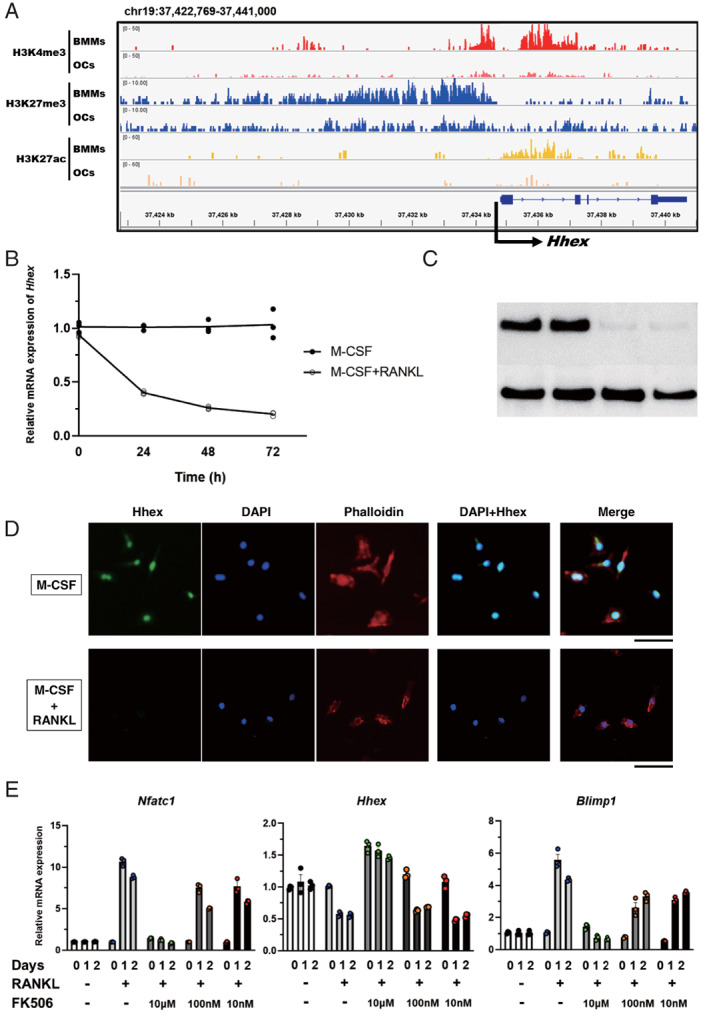
Hhex gene expression is downregulated in an epigenetic manner during osteoclastogenesis. (*A*) Histone modification of *Hhex* in BMMs and osteoclasts analyzed by ChIP‐seq of trimethylation of lysine 4 (H3K4me3) and lysine 27 (H3K27me3), as well as acetylation (H3K27ac) of histone H3. (*B*) Time course of changes in *Hhex* mRNA expression in BMMs cultured with M‐CSF or M‐CSF and RANKL. (*C*) Suppression of Hhex protein expression in response to RANKL stimulation as determined by immunoblot analysis. (*D*) Immunocytochemistry of BMMs cultured with M‐CSF or M‐CSF/RANKL for 2 days. Scale bar = 50 μm. (*E*) RANKL‐induced suppression of *Hhex* is mediated by the NFATc1‐Blimp1 axis. Time course of changes in *Nfatc1* (left), *Hhex* (middle), and *Blimp1* (right) expression in BMMs treated with M‐CSF alone or M‐CSF and RANKL in the presence of the indicated concentrations of FK506 or DMSO. DMSO = dimethyl sulfoxide.

We first conducted a genomewide screen for transcription factors epigenetically regulated during osteoclast differentiation. To identify negative regulators of osteoclast differentiation, we focused on genes whose histone modifications at the *cis*‐regulatory element changed from a H3K4me3‐and‐H3K27me3 bivalent status to H3K27me3 monovalent status in response to RANKL stimulation; in addition, a reduction of H3K27ac modification was confirmed. *Hhex*, a homeodomain transcription factor originally identified as a gene highly expressed in hematopoietic stem cells, is downregulated upon differentiation. H3K27me3 modification at the *cis*‐regulatory element of *Hhex* was maintained in osteoclasts, while H3K4me3 and H3K27ac modifications were markedly reduced in mature osteoclasts compared with M‐CSF‐dependent bone marrow‐derived macrophages (BMMs) (Fig. 1*A*). Expression of *Hhex* mRNA in BMMs, as detected by real‐time PCR, was decreased in a time‐dependent manner by RANKL stimulation (Fig. 1*B*). Protein levels of Hhex were also markedly decreased after 24 hours of RANKL stimulation, as shown in Fig. 1*C* by Western blotting. Immunocytochemistry with an anti‐Hhex antibody demonstrated that Hhex is mainly localized in the nuclei of BMMs, and its fluorescence intensity was reduced following RANKL stimulation (Fig. 1*D*). These results show that Hhex is down regulated in an epigenetic manner during RANKL‐induced osteoclastogenesis.

To determine whether the repression of Hhex by RANKL was dependent on NFATc1, we examined the effect of FK506, a calcineurin inhibitor that blocks the nuclear translocation and autoamplification of NFATc1.^(^ ^17^ ^)^ As shown in Fig. 1*E*, FK506 attenuated the RANKL‐induced reduction of *Hhex* expression in a dose‐dependent manner. Previously, B‐lymphocyte‐induced maturation protein (Blimp)1 (also known as PR domain zinc finger protein 1, PRDM1) was reported to stimulate osteoclast differentiation by downregulating negative regulators such as Irf8, MafB, and Bcl6; notably, Hhex was also negatively regulated by Blimp1.^(^ ^9^, ^10^ ^)^ FK506 dose‐dependently suppressed *Blimp1* and *Nfatc1* induction by RANKL, which was inversely correlated with *Hhex* expression (Fig. 1*E*). These results suggest that *Hhex* is negatively regulated by Blimp1 in an NFATc1‐dependent manner.

### *Hhex* is a negative regulator of osteoclastogenesis

**Fig. 2:**
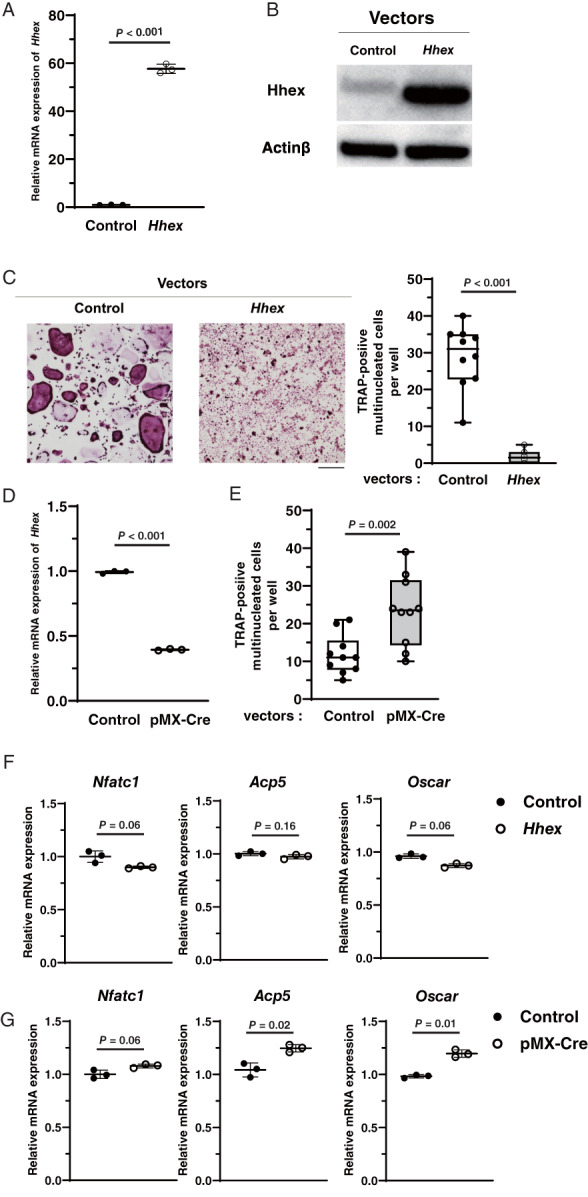
Hhex is a negative regulator of osteoclastogenesis. (*A*) mRNA expression of *Hhex* analyzed by real‐time PCR after transfection with pMX‐IRES‐Puro or pMX‐IRES‐Puro‐Hhex. (*B*) Immunoblot analysis of Hhex protein levels in BMMs after transfection with pMX‐IRES‐Puro and pMX‐IRES‐Puro‐Hhex. (*C*) Inhibition of RANKL‐induced TRAP‐positive MNC formation by retrovirus‐mediated overexpression of *Hhex*. (left) TRAP staining. Cultures were performed at least five times and representative pictures are shown. (right) Number of MNCs per well. Data represent the mean ± SD (*n* = 10). Scale bar = 100 μm. (*D*) Empty vectors or retrovirus vectors encoding Cre recombinase were infected into BMMs from *Hhex* ^flox/flox^ mice, and expression of *Hhex* mRNA was analyzed by real‐time PCR. (*E*) Number of TRAP‐positive MNCs per well after treatment with M‐CSF and RANKL for 3 days (*n* = 10). (*F*) mRNA expression levels of *Nfatc1*, *Acp5*, and *Oscar* when RANKL was administered to BMMs transfected with pMX‐IRES‐Puro and pMX‐IRES‐Puro‐Hhex. (*G*) mRNA expression of *Nfatc1*, *Acp5*, and *Oscar* when RANKL was administered to BMMs from Hhex^flox/flox^ mice transfected with pMX‐IRES‐Puro or pMX‐IRES‐Puro‐Cre. Control = pMX‐IRES‐Puro (Mock); *Hhex*, pMX‐IRES‐Puro‐Hhex (Hhex overexpression); MNC = multinucleated cell; pMX‐Cre = pMX‐IRES‐Puro‐Cre.

To investigate the role of Hhex in osteoclastogenesis, *Hhex* was overexpressed in BMMs using the retroviral vector pMX‐Hhex‐IRES‐Puro. BMMs infected either by pMX‐Hhex‐IRES‐Puro or a control vector pMX‐IRES‐Puro were treated with RANKL. Overexpression of *Hhex* in pMX‐Hhex‐IRES‐Puro–infected BMMs was confirmed by quantitative reverse transcription PCR (RT‐qPCR) and immunoblotting (Fig. 2*A*,*B*). As shown in Fig. 2*C*, *Hhex* overexpression markedly inhibited RANKL‐induced osteoclast differentiation from BMMs. Negative regulation of osteoclastogenesis by *Hhex* overexpression was further confirmed using another retroviral vector, pMX‐IRES‐Puro‐Hhex–enhanced green fluorescent protein (EGFP) (data not shown). Conversely, the effect of *Hhex* suppression on in vitro osteoclastogenesis was examined using *Hhex* ^fl/fl^ mice, which contain *loxP* sites flanking exon 2 of the *Hhex* gene. When BMMs obtained from *Hhex* ^fl/fl^ mice (*Hhex* ^fl/fl^ BMMs) were infected with pMx‐Cre retrovirus, expression of *Hhex* was significantly reduced (Fig. 2*D*). RANKL and M‐CSF treatment generated higher number of osteoclasts from *Hhex* ^fl/fl^ BMMs infected with pMx‐Cre virus compared with those infected with control virus (Fig. 2*E*). These results suggest that Hhex negatively regulates RANKL‐induced osteoclastogenesis from BMMs. Interestingly, there was no significant difference in the expression of *Nfatc1* or osteoclast marker genes when RANKL was stimulated in Hhex‐overexpressing BMMs or Hhex‐depleted BMMs (Fig. 2*F*,*G*).

### Low‐bone‐mass phenotype induced by *Hhex* deletion

**Fig. 3:**
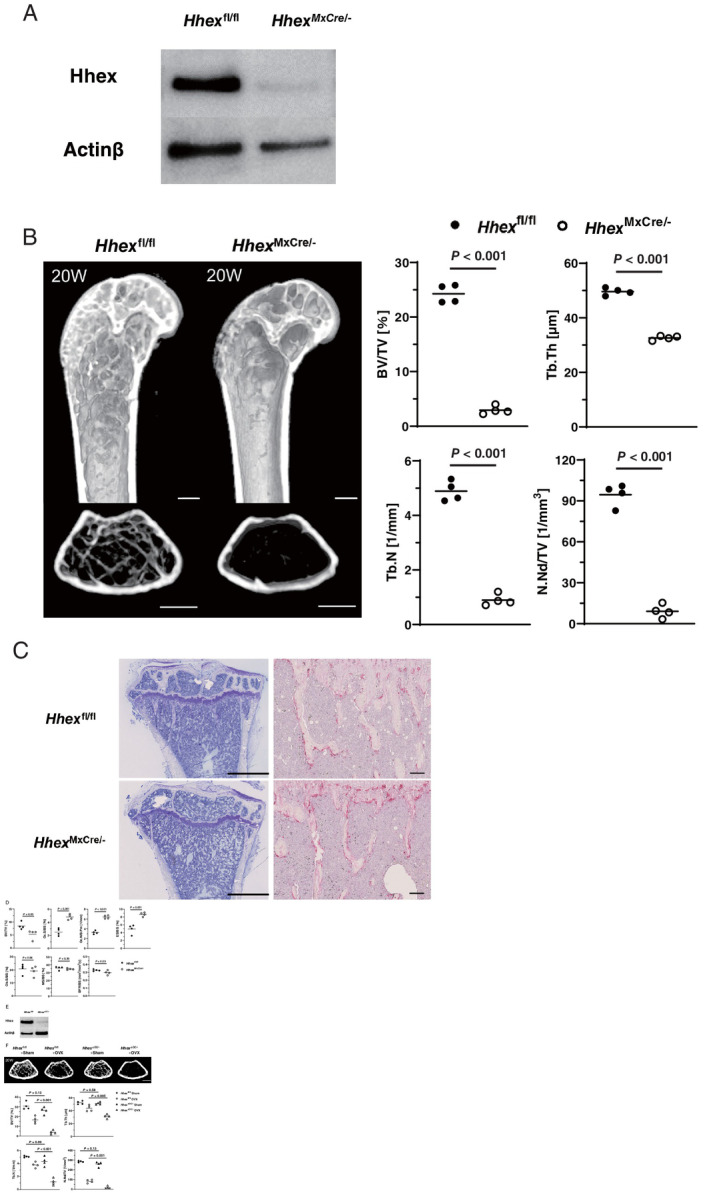
Low‐bone‐mass phenotypes induced by Hhex deletion. (*A*) Immunoblot analysis of Hhex protein levels in BMMs from *Hhex* ^flox/flox^ and *Hhex* ^MxCre/−^ mice. (*B*) (left) μCT analysis of femurs of *Hhex* ^flox/flox^ (*Hhex* ^fl/fl^) and Mx‐1^Cre/‐^ *Hhex* ^flox/flox^ (*Hhex* ^*MxCre*/−^) mice. pIpC injections (12 .5 μg/g of body weight) were administered to 12‐week‐old males in each group, which were euthanized 8 weeks after injection (*n* = 4 per group). Longitudinal (upper) and axial views (lower) of the metaphyseal region of mice. Scale bars = 500 μm. (right) μCT‐based parameters of the metaphyseal region. (*C*) Histological analysis of the proximal tibia of *Hhex* ^flox/flox^ and *Hhex* ^*MxCre*/−^. pIpC injections (12.5 μg/g of body weight) were administered to 20‐week‐old male mice in each group, which were euthanized after 8 weeks. (left) Toluidine blue staining. Scale bars = 1 mm. (right) TRAP staining. Scale bars = 100 μm. (*D*) Histomorphometric analysis of tibias from 20‐week‐old mice (*n* = 4 in each group). Parameters for osteoclastic bone resorption and osteoblastic bone formation in the bone morphometric analysis of *Hhex* ^flox/flox^ and *Hhex* ^MxCre/−^ mice. (*E*) Immunoblot analysis of Hhex protein levels in BMMs obtained from *Hhex* ^flox/flox^ and *CtsK* ^Cre/‐^ *Hhex* ^flox/flox^ (*Hhex* ^ΔOC/−^) mice. (*F*) μCT analysis of the femurs of *Hhex* ^flox/flox^ (*Hhex* ^fl/fl^) and *CtsK* ^Cre/−^ *Hhex* ^flox/flox^ (*Hhex* ^ΔOC/−^) mice. Twelve‐week‐old female mice were subjected to OVX or sham surgery and euthanized 8 weeks later (*n* = 4 per group). Axial views of the metaphyseal region of mice. Scale bar = 500 μm. μCT‐based parameters of the metaphyseal region. BFR/BS = bone formation rate per bone surface; BV/TV = bone volume per tissue volume; ES/BS = eroded surface per bone surface; MS/BS = mineralized surface per bone surface; N.Nd/TV = number of nodules per tissue volume; Oc.N/B.Pm = osteoclast number per bone perimeter; Ob.S/BS = osteoblast surface per bone surface; Oc.S/BS = osteoclast surface per bone surface; OVX = ovariectomy; Tb.N = trabecular number; Tb.Th = trabecular bone thickness.

We next analyzed the effect of *Hhex* deletion in vivo. Because mice with homozygous disruption of *Hhex* are embryonic lethal,^(^ ^18^, ^19^, ^20^ ^)^ we generated conditional knockout mice using the Cre‐loxP system. We crossed *Hhex* ^fl/fl^ mice with a transgenic line expressing Cre recombinase from a type I interferon inducible promoter (Mx1‐Cre), in which Cre recombinase is induced in response to treatment with pIpC (referred to as *Hhex* ^MxCre/−^ mice). Immunoblotting confirmed that Hhex protein expression was almost completely diminished in BMMs from *Hhex* ^MxCre/−^ mice treated with pIpC (Fig. 3*A*). In vitro osteoclast differentiation was increased when *Hhex* was deleted (Fig. S1 *A*). To investigate the effect of *Hhex* deletion in vivo, pIpC (12.5 μg/g of body weight) was injected intraperitoneally into 12‐week‐old male *Hhex* ^MxCre/−^ mice and *Hhex* ^fl/fl^ mice, which were euthanized 8 weeks after the injection to analyze skeletal tissues. μCT analysis of mouse femurs showed that *Hhex* ^MxCre/−^ mice treated with pIpC exhibited a marked reduction in trabecular bone volume, trabecular thickness, trabecular number, and the number of bone nodules compared with *Hhex* ^fl/fl^ mice (Fig. 3*B*). The same results were observed in female mice (Fig. S2). TRAP staining revealed an increase in osteoclasts on the bone surface of proximal tibia in *Hhex* ^MxCre/−^ mice as compared with *Hhex* ^fl/fl^ mice (Fig. 3*C*). Bone morphometric analysis demonstrated a reduction of bone volume associated with an increase in osteoclast number and an increase in the indicators of bone resorption. Bone formation parameters were slightly decreased in *Hhex* ^MxCre/−^ mice with no significant difference (Fig. 3*D*).

Because induction of the *Mx1* promoter by pIpC is not restricted to osteoclast‐lineage BMMs, we also generated *Hhex* conditional knockout mice by crossing *Hhex* ^fl/fl^ mice with cathepsin K‐Cre knock‐in mice, in which the Cre recombinase gene is inserted into the *cathepsin K* locus and expressed in osteoclast‐lineage cells (referred to as *Hhex* ^ΔOC/**−**^ mice). A significant reduction of *Hhex* was observed in BMMs from *Hhex* ^ΔOC/**−**^ mice after treatment with RANKL and M‐CSF (Fig. 3*E*), and osteoclast differentiation was increased when *Hhex* was deleted (Fig. S1 *B*). Because we observed only marginal effects by *Hhex* deletion on bone mass at baseline (Fig. S3 *A*–*C*), 12‐week‐old female *Hhex* ^ΔOC/**−**^ and control *Hhex* ^fl/fl^ mice were subjected to ovariectomy, and euthanized after 8 weeks. μCT analysis showed a significant reduction of trabecular bone volume, trabecular thickness, trabecular number, and the number of bone nodules in ovariectomized *Hhex* ^ΔOC/**−**^ mice compared with ovariectomized control *Hhex* ^fl/fl^ mice, as shown in Fig. 3*F*.

These results clearly demonstrate the negative regulatory role of Hhex in osteoclastogenesis both in vitro and in vivo.

### Mechanisms of action of Hhex in osteoclast differentiation

**Fig. 4:**
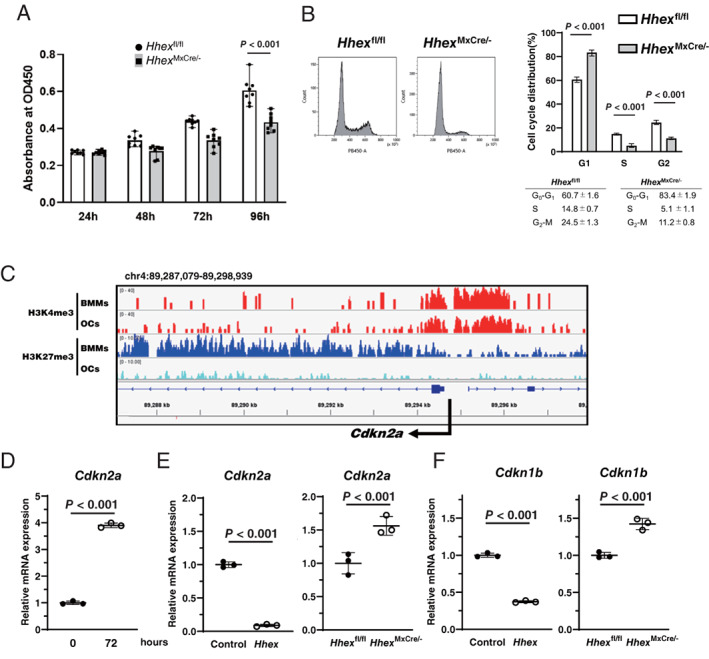
Hhex regulates the cell cycle of BMMs by inhibiting cyclin‐dependent kinase inhibitor expression. (*A*) Cell proliferation rate was assessed by WST‐8 assay. A significant reduction of cell proliferation was observed in BMMs from *Hhex* conditional knockout (*Hhex* ^MxCre/−^) mice compared with *Hhex* ^flox/flox^ (*Hhex* ^fl/fl^) mice (*n* = 8). (*B*) Cell cycle analysis of BMMs from *Hhex* ^flox/flox^ and *Hhex* ^MxCre/−^ mice using flow cytometry (*n* = 4). (*C*) Histone modification at the *cis*‐regulatory element of *Cdkn2a*, as analyzed by ChIP‐seq in BMMs and osteoclasts. H3K27me3 modification at the *cis*‐regulatory element of *Cdkn2a* gene was decreased in mature osteoclasts compared with BMMs, while the H3K4me3 modification did not appear to differ between BMMs and osteoclasts. (*D*) mRNA expression of *Cdkn2a* was increased in BMMs by RANKL treatment for 72 hours. (*E*) Negative regulation of *Cdkn2a* by *Hhex* in BMMs. (left) Retrovirus (pMX‐IRES‐Puro‐Hhex)‐induced overexpression of *Hhex* suppressed *Cdkn2a* expression in BMMs. (right) Increased expression of *Cdkn2a* in BMMs from Mx‐1^Cre/−^ *Hhex* ^flox/flox^ mice compared with those from *Hhex* ^flox/flox^ mice. (*F*) Negative regulation of *Cdkn1b* by Hhex. (left) Retrovirus‐induced overexpression of *Hhex* suppressed *Cdkn1b* expression in BMMs. (right) Increased expression of *Cdkn1b* in BMMs from Mx‐1^Cre/−^ *Hhex* ^flox/flox^ mice compared with those from *Hhex* ^flox/flox^ mice. Control = pMX‐IRES‐Puro; *Hhex* = pMX‐IRES‐Puro‐Hhex; *Hhex* ^fl/fl^ = *Hhex* ^flox/flox^; *Hhex* ^ΔMxCre/−^ = Mx‐1^Cre/−^ *Hhex* ^flox/flox^.

We next addressed the mechanism of how Hhex negatively regulates RANKL‐induced osteoclast differentiation. Cell‐cycle regulation is critical for osteoclast differentiation, and previous studies identified cell cycle–arrested quiescent osteoclast precursors as committed osteoclast precursors.^(^ ^21^, ^22^ ^)^ Therefore, we analyzed the effect of *Hhex* deletion on cell proliferation and cell‐cycle distribution in BMMs. Proliferation of BMMs, as determined by WST‐8, was reduced in *Hhex* ^MxCre/−^ BMMs compared with *Hhex* ^fl/fl^ BMMs (Fig. 4*A*). Analyses of cell‐cycle distributions by flow cytometry showed that *Hhex* deletion in BMMs reduced the proportion of cells at S phase from (mean ± standard deviation [SD]) 14.8% ± 0.7% to 5.1% ± 1.1%, and increased those at G1 phase from 60.7% ± 1.6% to 83.4% ± 1.9%, indicating that Hhex regulated cell cycle status and its deletion induced G1 cell‐cycle arrest in BMMs (Fig. 4*B*).

The mammalian cell cycle is regulated by the balance between cyclin‐dependent kinases and cyclin‐dependent kinase inhibitors (CDKIs), and the terminal differentiation is usually coupled with permanent exit from the cell cycle.^(^ ^23^, ^24^, ^25^ ^)^ Hhex reportedly binds to the *Cdkn2a* locus and directly interacts with PRC2 to enable H3K27me3‐mediated epigenetic repression, and targeted deletion of *Hhex* stimulates *Cdkn2a* expression and suppresses self‐renewal of hematopoietic stem cells.^(^ ^26^ ^)^ Therefore, we next examined the effects of *Hhex* overexpression and suppression on *Cdkn2a* expression in BMMs. ChIP‐seq analysis of BMMs and osteoclasts demonstrated that H3K27me3 modification at the *cis*‐regulatory element of *Cdkn2a* was decreased in mature osteoclasts compared with BMMs, whereas H3K4me3 modification did not appear to differ between BMMs and osteoclasts (Fig. 4*C*), and expression of *Cdkn2a* was increased after 72 hours of RANKL stimulation (Fig. 4*D*). Overexpression of *Hhex* suppressed RANKL‐induced *Cdkn2a* expression, which was increased by *Hhex* deletion (Fig. 4*E*). In addition to Cdkn2a, there are at least two distinct families of CDKIs in mammalian cells: the p21/p27 and p16/p18 families. Similar to Cdkn2a, expression of Cdkn1b, which encodes p27, was suppressed by Hhex overexpression and increased by *Hhex* deletion (Fig. 4*F*), whereas expression of *Cdkn1a* (*p21*) and *Tp53* was not affected by Hhex (Fig. S4).

## Discussion

Herein we showed that Hhex, a member of the tinman family of homeodomain‐containing transcription factors, is downregulated by RANKL‐mediated epigenetic modification in BMMs and plays an important role in osteoclast development by regulating cell‐cycle progression. Histone modification of the *cis*‐regulatory element of *Hhex* changed from a H3K4me‐and‐H3K27me3 bivalent status to H3K27me3 monovalent status in response to RANKL stimulation, and H3K27ac modification was reduced, which was correlated with a reduction of *Hhex* expression. These results suggest that *Hhex* expression was epigenetically silenced during osteoclast differentiation. RANKL‐induced inhibition of *Hhex* appears to be NFATc1 dependent because suppression of NFATc1 activation by FK506 restored inhibition.

Hhex, a relatively unusual homeodomain protein, exists as a homo‐oligomer^(^ ^27^ ^)^ and is highly conserved between species.^(^ ^28^ ^)^ Hhex was first identified in avian and human hematopoietic cells, whereby it was found to regulate cell development and differentiation by both transcriptional and posttranscriptional mechanisms.^(^ ^29^, ^30^ ^)^ Hhex is expressed in early hematopoietic progenitors of all lineages except T‐cell lineages, especially prevalent in hematopoietic stem cells, and downregulated upon terminal differentiation of these cells.^(^ ^29^, ^31^ ^)^ During embryonic development, Hhex is essential for forebrain, liver, and thyroid development; thus, targeted disruption of *Hhex* in mice leads to embryonic lethality.^(^ ^18^, ^19^, ^20^ ^)^ Blastocyst compensation in *Hhex* ^−/−^; *Rag1* ^−/−^ chimeric mice or conditional deletion of *Hhex* in the hematopoietic system showed that Hhex is dispensable for maintenance of hematopoietic stem cells, but critical for early lymphoid specification; in particular, development of B cell lineages.^(^ ^32^, ^33^, ^34^, ^35^ ^)^ However, the role of Hhex in skeletal homeostasis and osteoclast‐lineage cells has not been clarified.

To analyze the role of Hhex in osteoclast‐lineage cells, we first performed in vitro experiments and found that overexpression of *Hhex* in BMMs almost completely suppressed RANKL‐induced osteoclast development, whereas its deletion resulted in increased osteoclastogenesis. The role of Hhex in osteoclastogenesis was further confirmed by in vivo experiments using *Hhex* ^MxCre/−^ mice, in which *Hhex* is inducibly deleted in hematopoietic cells by pIpC administration. *Hhex* was efficiently deleted in BMMs of *Hhex* ^MxCre/−^ mice, which exhibited reduced bone mass due to increased numbers of osteoclasts. Decreased bone mineral density was also observed in Hhex^ΔOC/−^ mice. These results demonstrate that Hhex is a negative regulator of osteoclast‐lineage cells both in vitro and in vivo. It should be noted that the skeletal phenotypes of *Hhex* ^MxCre/−^ mice and *Hhex* ^ΔOC/**−**^ mice are somehow different (Fig. 3*A*,*E*). The exact reason for this difference is unknown, but we speculate that it may be because not all BMMs differentiate into mature osteoclasts in vivo. Furthermore, it is possible that Mx1‐Cre affects other types of hematopoietic cells than monocyte/macrophage‐lineage cells, which indirectly affect osteoclast differentiation.

We next addressed the mechanism how Hhex negatively regulates osteoclastogenesis. It was previously reported that overexpression of Hhex in hematopoietic cells induced murine lymphoid neoplasms,^(^ ^36^ ^)^ and *HHEX* is expressed at high levels in human acute myeloid leukemia cells and required for the development of myeloid leukemia driven by the oncogenic fusion protein mixed lineage leukemia‐eleven nineteen leukemia (MLL‐ENL).^(^ ^26^ ^)^ Moreover, the ability of Hhex to promote myeloid progenitor expansion and acute myeloid leukemogenesis arose from its function of repressing the tumor suppressor gene *Cdkn2a*, by binding to the *Cdkn2a* locus and directly interacts with PRC2 to enable H3K27me3‐mediated epigenetic repression.^(^ ^26^ ^)^ *Cdkn2a* encodes two CDKIs, p16^INK4a^ and p19^ARF^, suggesting that Hhex is involved in the regulation of cell cycle progression.

The critical role of cell cycle regulation in osteoclast development has been reported in previous studies. Okahashi and colleagues^(^ ^37^ ^)^ reported that RANKL induced transient expression of p21 and p27, and suppression of these two CDKIs by antisense oligonucleotides strongly inhibited osteoclast differentiation. Sankar and colleagues^(^ ^38^ ^)^ reported that mice with double knockout of CDKIs p21 and p27 developed osteopetrosis, with fewer osteoclasts exhibiting lower TRAP activity and abnormal cell morphology present in long bone. Kwon and colleagues^(^ ^39^ ^)^ reported that M‐CSF deprivation induced cell cycle arrest at the G0/G1 phase and promoted osteoclast differentiation. We previously reported that Cdk6 levels are downregulated in osteoclast precursors by RANKL in a NF‐κB–dependent manner, and overexpression of Cdk6 suppressed osteoclastogenesis.^(^ ^40^ ^)^ Mizoguchi and colleagues^(^ ^21^ ^)^ and Muto and colleagues^(^ ^22^ ^)^ identified cell‐cycle–arrested quiescent osteoclast precursors along bone surfaces that rapidly and directly differentiated into mature osteoclasts in response to RANKL. Collectively, these results suggest that RANKL promotes osteoclast differentiation by inducing cell cycle arrest in BMMs. Therefore, we hypothesized that Hhex regulates osteoclast differentiation by modulating RANKL‐induced cell cycle arrest in BMMs. Analysis of cell‐cycle distribution by flow cytometry showed that *Hhex* deletion in BMMs reduced the proportion of cells at S phase and increased those at G1 phase. ChIP‐seq analysis of BMMs and osteoclasts demonstrated that the H3K27me3 modification at the *cis*‐regulatory element of *Cdkn2a* was decreased in mature osteoclasts as compared with BMMs, whereas the H3K4me3 modification did not appear to differ between BMMs and osteoclasts, and expression of *Cdkn2a* was increased in osteoclasts (Fig. 4*D*). Overexpression of Hhex suppressed RANKL‐induced *Cdkn2a* expression, which was increased by *Hhex* deletion (Fig. 4*E*). In addition to *Cdkn2a*, expression of *Cdkn1b* was negatively regulated by Hhex. These results led us to conclude that Hhex positively regulates the cell‐cycle progression of BMMs by downregulating *Cdkn2a* and *Cdkn1b*, and downregulation of Hhex is required for RANKL‐induced cell‐cycle arrest and osteoclastogenesis. Farr and colleagues^(^ ^41^ ^)^ recently reported that *p16* ^Ink4a^‐expressing senescent cells in the bone microenvironment are implicated in age‐related bone loss, which was ameliorated by inducible elimination of *p16* ^Ink4a^‐expressing cells. Li and colleagues^(^ ^42^ ^)^ demonstrated that osteoclast differentiation was suppressed in *p16* ^Ink4a^‐deficient mice, which were resistant to ovariectomy‐induced bone loss. In addition, a single nucleotide polymorphism of *HHEX*, rs2497306, was associated with human aging by regulation of serum dehydroepiandrosterone sulfate levels.^(^ ^43^ ^)^ These results suggest that the Hhex‐Cdkn2a axis may be involved in age‐related bone loss. However, the role of Hhex in regulation of *Cdkn2a* and *Cdkn1b* in bone cells other than osteoclasts remains unknown, and further studies are required to elucidate the role of Hhex in age‐related bone loss.

There are several limitations of this study. First, the mechanism of Hhex‐induced downregulation of *Cdk2a* and *Cdk1b* remains unclear. Reportedly, Hhex interacts with members of the Groucho/transducin‐like enhancer of split co‐repressor protein family and may recruit these proteins to a subset of target promoters to bring about transcriptional repression.^(^ ^44^ ^)^ However, future studies are required to identify the partners of Hhex and global landscape of Hhex‐regulated gene expression in BMMs. Second, it remains unclear how cell‐cycle regulation affects RANKL‐induced osteoclast differentiation. It is widely recognized that there is a temporal coupling between cell‐cycle arrest and terminal differentiation, although cell‐cycle arrest per se is not sufficient for cell differentiation.^(^ ^45^ ^)^ Muto and colleagues^(^ ^22^ ^)^ identified cell cycle‐arrested quiescent osteoclast precursors along bone surfaces as committed osteoclast precursors. Motiur Rahman and colleagues^(^ ^46^ ^)^ reported that RANKL stimulates DNA synthesis and cell proliferation of BMMs during the early phase of osteoclastogenesis, followed by G1 arrest in the latter half of differentiation. Therefore, it is possible that Hhex‐mediated suppression of CDKIs stimulates the proliferation of BMMs at the early stage, whereas subsequent downregulation of Hhex induces cell‐cycle arrest. Further studies are required to identify the mechanism by which cell‐cycle arrest affects osteoclast differentiation. Small sample size in conditional knockout mice is also considered as limitation of this study. Last, the clinical relevance of the present results should be further clarified. It is possible that the Hhex‐Cdkn2a axis is involved in age‐related bone loss by regulating cellular senescence. Interestingly, genomewide association studies demonstrated the association of single nucleotide polymorphisms of *HHEX* and *CDKN2a* genes with type 2 diabetes mellitus.^(^ ^47^, ^48^ ^)^ It has been well established that individuals with type 2 diabetes are at an increased risk of osteoporotic fractures, and it is possible that the modulation of *HHEX* gene expression may play a role in bone fragility in these patients. Accordingly, it is of particular interest to investigate the relationship between *HHEX* expression levels and bone mineral density in patients with type 2 diabetes in a clinical setting.

In conclusion, we demonstrated that the homeodomain‐containing transcription factor Hhex negatively regulates RANKL‐induced osteoclastogenesis by modulating cell‐cycle progression. This study highlights the regulation of Hhex levels as a potential strategy to inhibit osteoporosis‐induced bone loss and fractures.

## Conflicts of Interest

The authors state that they have no conflicts of interest.

### Peer Review

The peer review history for this article is available at https://publons.com/publon/10.1002/jbm4.10608.

## Supporting information


**Fig. S1.** (*A*) Increased osteoclast differentiation in response to M‐CSF and RANKL in BMMs from *Hhex* ^*MxCre*/−^ mice compared with *Hhex* ^flox/flox^ mice. (Left) TRAP staining (Right) Number of TRAP‐positive multinucleated cell (MNC) per well (*n* = 10). Bar = 500 μm. *Hhex* ^ΔMxCre/−^; Mx‐1^Cre/−^ *Hhex* ^flox/flox^. (*B*) Increased osteoclast differentiation in response to M‐CSF and RANKL in BMMs from *Hhex* ^ΔOC/−^ mice compared with *Hhex* ^flox/flox^ mice. (Left) TRAP staining. (Right) Number of TRAP‐positive MNC per well (*n* = 10). Bar = 500 μm. *Hhex* ^ΔOC/−^; *CtsK* ^Cre/−^ *Hhex* ^flox/flox^.

**Fig. S2.** (*A*) We performed μCT analysis of femurs in females as well as males *Hhex* ^flox/flox^ (*Hhex* ^fl/fl^) and Mx‐1^Cre/−^ *Hhex* ^flox/flox^ (*Hhex* ^MxCre/−^) mice. pIpC injections (12.5 μg/g of body weight) were administered to 12‐week‐old females in each group, which were euthanized 8 weeks after injection (*n* = 4 per group). μCT‐based parameters of the metaphyseal region. BV/TV, bone volume per tissue volume; Tb.Th, trabecular bone thickness; Tb.N, trabecular number; N.Nd/TV, number of nodules per tissue volume.

**Fig. S3.** (*A*) Microcomputed tomography (μCT) analysis of the femurs of *Hhex* ^flox/flox^ mice and CtsK^Cre/−^ *Hhex* ^flox/flox^ (*Hhex* ^ΔOC/−^) mice at baseline (10‐week‐old female). Representative μCT images of axial views of the metaphyseal region of *Hhex* ^flox/flox^ mice and *Hhex* ^ΔOC/−^ mice are shown. (*B*) μCT‐based parameters of the metaphyseal region of 10‐week‐old female. BV/TV, bone volume per tissue volume; Tb.Th, trabecular bone thickness; Tb.N, trabecular number; N.Nd/TV, number of nodules per tissue volume. *Hhex* ^fl/fl^; *Hhex* ^flox/flox^, *Hhex* ^ΔOC/−^; CtsK^Cre/−^ *Hhex* ^flox/flox^. (*C*) μCT‐based parameters of the metaphyseal region of 10‐week‐old male. BV/TV, bone volume per tissue volume; Tb.Th, trabecular bone thickness; Tb.N, trabecular number; N.Nd/TV, number of nodules per tissue volume. *Hhex* ^fl/fl^; *Hhex* ^flox/flox^, *Hhex* ^ΔOC/−^; CtsK^Cre/−^ *Hhex* ^flox/flox^.

**Fig. S4.** (*A*) Effects of *Hhex* overexpression or depletion on mRNA expression of *Cdkn1a* in BMMs. (Left) Retrovirus‐induced overexpression of *Hhex* showed no significant effect on *Cdkn1a* expression in BMMs. (Right) No significant difference in *Cdkn1b* or *Tp53* expression was observed between BMMs from *Hhex* ^flox/flox^ mice and *Hhex* ^MxCre/−^ mice. Control, pMX‐IRES‐Puro (Mock); *Hhex*, pMX‐IRES‐Puro‐Hhex (Hhex overexpression). *Hhex* ^fl/fl^; *Hhex* ^flox/flox^, *Hhex* ^ΔMxCre/−^; Mx‐1^Cre/‐^ *Hhex* ^flox/flox^. (*B*) Effects of *Hhex* overexpression or depletion in BMMs on mRNA expression of *Tp53* in BMMs. (Left) Retrovirus‐induced overexpression of *Hhex* showed no significant effect on *Tp53* expression in BMMs. (Right) No significant difference in *Tp53* expression was observed between BMMs from *Hhex* ^flox/flox^ mice and *Hhex* ^MxCre/−^ mice. Control, pMX‐IRES‐Puro (Mock); *Hhex*, pMX‐IRES‐Puro‐Hhex (Hhex overexpression). *Hhex* ^fl/fl^; *Hhex* ^flox/flox^, *Hhex* ^ΔMxCre/−^; Mx‐1^Cre/‐^ *Hhex* ^flox/flox^.
