# Supplementary material for: Transcription Factor Hematopoietically Expressed Homeobox Protein (Hhex) Negatively Regulates Osteoclast Differentiation by Controlling Cyclin‐Dependent Kinase Inhibitors
Source: JBMR Plus. 2022 Feb 14;6(4):e10608. doi: 10.1002/jbm4.10608 (PMC9009129; doi:10.1002/jbm4.10608)

Supplemental material

Figure S1

A

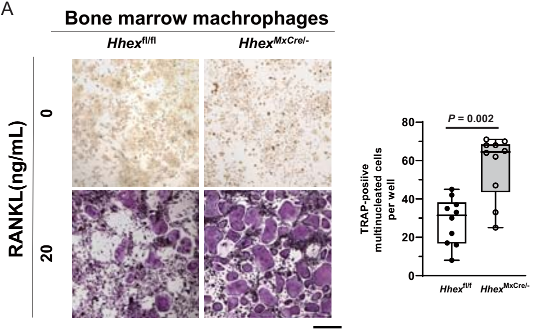

B

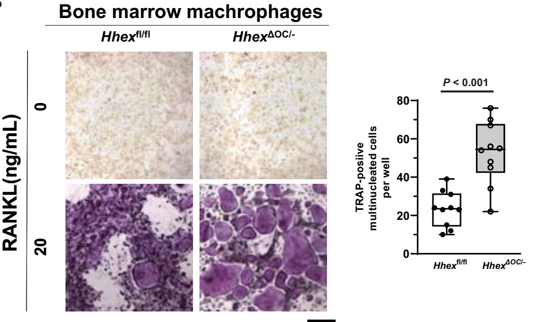

Supplement: Supplementary file 1 — Fig. S1. (A) Increased osteoclast differentiation in response to M‐CSF and RANKL in BMMs from Hhex MxCre/− mice compared with Hhex flox/flox mice. (Left) TRAP staining (Right) Number of TRAP‐positive multinucleated cell (MNC) per well (n = 10). Bar = 500 μm. Hhex ΔMxCre/−; Mx‐1Cre/− Hhex flox/flox. (B) Increased osteoclast differentiation in response to M‐CSF and RANKL in BMMs from Hhex ΔOC/− mice compared with Hhex flox/flox mice. (Left) TRAP staining. (Right) Number of TRAP‐positive MNC per well (n = 10). Bar = 500 μm. Hhex ΔOC/−; CtsK Cre/− Hhex flox/flox. [file JBM4-6-e10608-s001.pdf]
