# Supplementary figures and images for: Transcription Factor Hematopoietically Expressed Homeobox Protein (Hhex) Negatively Regulates Osteoclast Differentiation by Controlling Cyclin‐Dependent Kinase Inhibitors
Source: JBMR Plus. 2022 Feb 14;6(4):e10608. doi: 10.1002/jbm4.10608 (PMC9009129; doi:10.1002/jbm4.10608)

# Figure S2

A

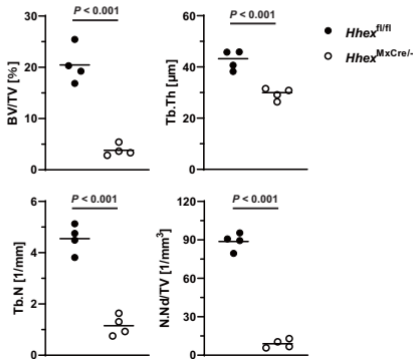

Supplement: Supplementary file 2 — Fig. S2. (A) We performed μCT analysis of femurs in females as well as males Hhex flox/flox (Hhex fl/fl) and Mx‐1Cre/− Hhex flox/flox (Hhex MxCre/−) mice. pIpC injections (12.5 μg/g of body weight) were administered to 12‐week‐old females in each group, which were euthanized 8 weeks after injection (n = 4 per group). μCT‐based parameters of the metaphyseal region. BV/TV, bone volume per tissue volume; Tb.Th, trabecular bone thickness; Tb.N, trabecular number; N.Nd/TV, number of nodules per tissue volume. [file JBM4-6-e10608-s002.pdf]

Figure S3

A

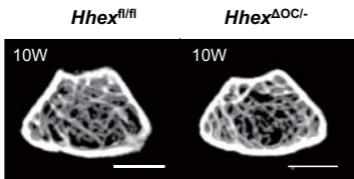

B

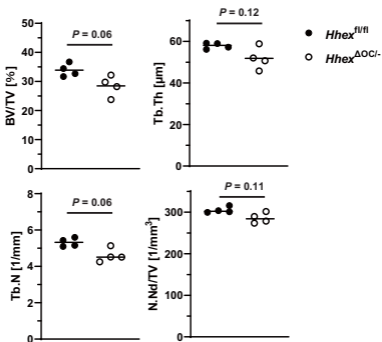

C

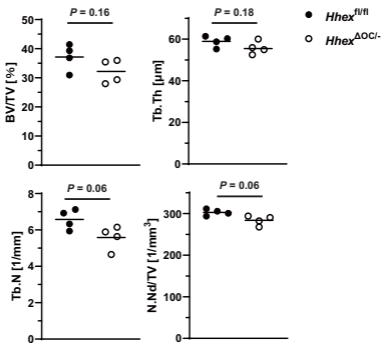

Supplement: Supplementary file 3 — Fig. S3. (A) Microcomputed tomography (μCT) analysis of the femurs of Hhex flox/flox mice and CtsKCre/− Hhex flox/flox (Hhex ΔOC/−) mice at baseline (10‐week‐old female). Representative μCT images of axial views of the metaphyseal region of Hhex flox/flox mice and Hhex ΔOC/− mice are shown. (B) μCT‐based parameters of the metaphyseal region of 10‐week‐old female. BV/TV, bone volume per tissue volume; Tb.Th, trabecular bone thickness; Tb.N, trabecular number; N.Nd/TV, number of nodules per tissue volume. Hhex fl/fl; Hhex flox/flox, Hhex ΔOC/−; CtsKCre/− Hhex flox/flox. (C) μCT‐based parameters of the metaphyseal region of 10‐week‐old male. BV/TV, bone volume per tissue volume; Tb.Th, trabecular bone thickness; Tb.N, trabecular number; N.Nd/TV, number of nodules per tissue volume. Hhex fl/fl; Hhex flox/flox, Hhex ΔOC/−; CtsKCre/− Hhex flox/flox. [file JBM4-6-e10608-s004.pdf]

# Figure S4

A

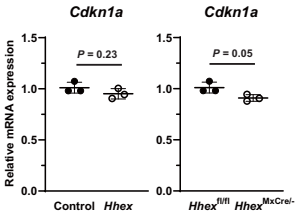

B

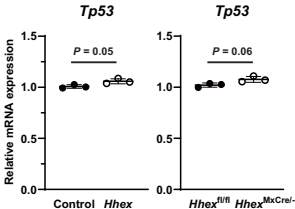

Supplement: Supplementary file 4 — Fig. S4. (A) Effects of Hhex overexpression or depletion on mRNA expression of Cdkn1a in BMMs. (Left) Retrovirus‐induced overexpression of Hhex showed no significant effect on Cdkn1a expression in BMMs. (Right) No significant difference in Cdkn1b or Tp53 expression was observed between BMMs from Hhex flox/flox mice and Hhex MxCre/− mice. Control, pMX‐IRES‐Puro (Mock); Hhex, pMX‐IRES‐Puro‐Hhex (Hhex overexpression). Hhex fl/fl; Hhex flox/flox, Hhex ΔMxCre/−; Mx‐1Cre/‐ Hhex flox/flox. (B) Effects of Hhex overexpression or depletion in BMMs on mRNA expression of Tp53 in BMMs. (Left) Retrovirus‐induced overexpression of Hhex showed no significant effect on Tp53 expression in BMMs. (Right) No significant difference in Tp53 expression was observed between BMMs from Hhex flox/flox mice and Hhex MxCre/− mice. Control, pMX‐IRES‐Puro (Mock); Hhex, pMX‐IRES‐Puro‐Hhex (Hhex overexpression). Hhex fl/fl; Hhex flox/flox, Hhex ΔMxCre/−; Mx‐1Cre/‐ Hhex flox/flox. [file JBM4-6-e10608-s003.pdf]
